# Supplementary material for: SUMOylation contributes to proteostasis of the chloroplast protein import receptor TOC159 during early development
Source: eLife. 2020 Dec 22;9:e60968. doi: 10.7554/eLife.60968 (PMC8497054; doi:10.7554/eLife.60968)
Supplement: Figure 1—source data 1. [file elife-60968-fig1-data1.docx]

CLUSTAL O(1.2.4) multiple sequence alignment

OsToc159 MAST-------------------------------------------------------- 4

SbToc159 MATT-------------------------------------------------------- 4

AtToc159 MDSKSVTPEPTN--------PFYASSGQSGKTYASVVAAAAAAAAD-------------K 39

PsToc159 MDSQTLS----------------SSSQ-FHEP-NNLLNGVNGHGSD------------SD 30

Solyc09g074940.1.1 MDSEEATFSPPAVSSSPGSSPINNSSS-NHTE-TENVSKINVEINDSDINSNSNSEGKSA 58

* :

OsToc159 -------TDADAAPVAPVLEEK---------------------------PPTPPPDGDEV 30

SbToc159 -------TDA-ADPVAPPVEEESAAPAAAEEEPPKKVEEAVATTDAAPVAPAPAPVEDET 56

AtToc159 EDGGAVSSAKELDSSSE--------------------------AVSGNSDKVGADDLSDS 73

PsToc159 SDDGFVSGEDEAEPSTPILVYDAKSTVQVKE----KEEESFDQESPRPIAKVTADDEDEA 86

Solyc09g074940.1.1 SDVTIVGGQQ---------------------------------ELPIPADP------DEG 79

.:

OsToc159 PSAPAA-AAAAEQ---------------PKVVEEEEVRLE----------GKGGGFGGQE 64

SbToc159 PAPAAA-AAAAEEESTKKVEAAAAE--EVEEDKEAPAKAE----------GVGGGHDVGE 103

AtToc159 E--KEKPNLVGDGKVSDEVDGSLKEDSTTPEATPKPEVVSGETIGVDDVSSLS------- 124

PsToc159 EEEEDDSQGVGLEKEGGG-GGGKDV----EEVKEDEVFVEANDKGFESVDSEGGDVVGEE 141

Solyc09g074940.1.1 T----LEKTIGEEKLDDSVVGSAEI----E----KPVSEVSMSEGVENVEALGGDVGGS- 126

. . .

OsToc159 VEVAG--DGEDGGEV--------------------EVAE--------AKDEGGGGEFAGG 94

SbToc159 VKEAE--EDDKGGNL--------------------GVAE--------AEKDGGGEELASE 133

AtToc159 ------------------------PKP-EAVSDGVGVVEENKKVKEDVEDIKDDGE---- 155

PsToc159 INNGLREEGDGGTEIETVRSDSVVVEPVSVDNSGVGVVENGDGVVDN-EKLTSGGDFVVD 200

Solyc09g074940.1.1 ------------------------------------------------------------ 126

OsToc159 DAKAASSLLAAAAEEEEE-------EEASNGELGEE--------DAYPA----------- 128

SbToc159 DGEAAPSGAEP--VPVVE-------SKSENGELGEG--------DPSLA----------- 165

AtToc159 --------------SKIE-----------NGSVD-------------------------- 164

PsToc159 SLRVNPLVD--GGVAVVGDEVKDEVSEIDGAVAPAPV---ASLDNSFEAIEKVGSRSVVD 255

Solyc09g074940.1.1 -------------VPVIGNSLPDSTDSDATKSLGTGIEGSEGNTEEFDSVDKLNSIEQVK 173

OsToc159 ----SSDAAVGEEKGELGEEPEEK-----------------AP----------ALAPEAN 157

SbToc159 ----FHDALEGDEKGELQKEEQQEDEE------------ERGA----------ALEVEVV 199

AtToc159 -----VDVKQASTDGESESKVKDVEEEDVGTKKDDEGESELGGKVDVDDKSDNVIEEEG- 218

PsToc159 EVGSSFE---TIEKGDEVV----VDDE------------VVGGDVEPSKVVDSGVEIEVD 296

Solyc09g074940.1.1 DNGGEVAVGAGLKEGEDRSTQEEVKET------------VEDEKMEPKEGGDRSIEEEVK 221

.*: : *

OsToc159 GA------A--------------ESDVEEKPEEDNEGEEVATGGGDDGELGME------- 190

SbToc159 DKVADDAEA--------------PVAAEKLEPEIEKGEEVGSGSGDGGELSDE------- 238

AtToc159 --VE--------LT-DKGDVIVNSSPVESVHVDVAKPGVVVVG-DAEGSEELKINADAET 266

PsToc159 DNVAHEQLSDVVLTEKAGDVVVDE----NVGVGAKPDEVVDIG-VDEGVAQR-------- 343

Solyc09g074940.1.1 ETVED------------------E----KIELQ---------G-GEDRSIQE-------- 241

. *

OsToc159 ----KEV----------DV----SAGAAEAPQPEDKVAPEAEANGDLGDKAE---EEASA 229

SbToc159 ----KEV----------EV----SPPSEEVAEPQDKVAPEA--NGELGDEKE---ESDDV 275

AtToc159 LEVANKFDQIGDDDSG-EFEPVSDKAIEEVEEKFTSESD-SIADSSKLESVDTSAVEPEV 324

PsToc159 -----QVSDIAPAEKGEEISEVVSQSLEAAED---------------------------- 370

Solyc09g074940.1.1 -----EVKEIVEDEKNEALTSVASSNLKEAEEPTSVIEESAIASSNLKESEEPTSVFEEV 296

:. . . . :

OsToc159 SAAVEVVEESNAPEEL-----LEKAV---------------------------------- 250

SbToc159 VA----LGGEEAPEES-----TNKDADG----------------DDVVALGGEEAPEES- 309

AtToc159 VAAES----GSEPKDVEKANGLEKGMTYAEVIKAASAVADNGTKEEESVLGGIV---DDA 377

PsToc159 ------------------------------EINIENRVVEG-GIESRVVEGGIESRVDDA 399

Solyc09g074940.1.1 AIASSNLKEAEEPTSVIEER----AIHSDDAEKLNKVVVEQ-PSESLLAET--------- 342

OsToc159 --------VSEANGVAAAVELAVEE-------------------------KLEDNKGEEE 277

SbToc159 -------TNKDADGD-DVVALGGDE----------------------APEESTKKDADVE 339

AtToc159 EEG----VKLNNKGDFVVDSSAIEA-------VNVDVAKPGVVVVGDVEVSELETDGNIP 426

PsToc159 VEGEVGSNVVEVEDGSNVDNVAEKDAVSNVDDAAEKDAVSNVDRVVEVEDESHV------ 453

Solyc09g074940.1.1 -----GSKKFTSEGDAVVDAIEVN-----V----SG---PGVAVVGDVDESKEV------ 379

.. . . .

OsToc159 EMEAKPEPVSGV------------------IPV-----------VVDDTS----SEMIAP 304

SbToc159 DEATKPEPPSEA------------------SPV-----------VLNDES----IEELAP 366

AtToc159 DVHNKFDPIGQGEGGEVELESDKATEEGG-GKLVSEGDSMVDSSVVDSVDADINVAEPGV 485

PsToc159 ---------GNTVEGEARSNADHVLQVEDET-------------HLDNAA----VGEAKS 487

Solyc09g074940.1.1 ---------EEHIEGTHDENVTSVNDVGETRQLIEEVAKM----TVDEVD----AQNPKP 422

::.

OsToc159 V---SAESAVEESTEKEQTVDDTSSEMIAHVSAESAVEES--------TEKEQTVESE-- 351

SbToc159 A---TADSVLEDSPEKEQNADA---------------------------------QTT-- 388

AtToc159 VVVGAAKEAVIKEDDKDDEVDKTI----------SNIEEPDDLTAAYDGNFELAVKE--- 532

PsToc159 NAD----RVVEVED--ETPLDNAA-----VGEAESNVDP--AVKVEDDTRFDNGAEGE-- 532

Solyc09g074940.1.1 VVD----DTVATAE--SKPVDNIV----------------------GAGKLDSGVVQTGD 454

.: . *

OsToc159 ASESVEIVGVEKPTEDESN----VDGGASSV--VSRELAPEETKENNVGQEDEGVAEVID 405

SbToc159 ASEVVEDVGVDKPTEVENV----AAPSADGI--LSRELAPESSNENKGADEIEGVTEVVD 442

AtToc159 ----ISEAAKVEPDEPKVGVEVEELPVSESLKVGSVDAEEDSIPAAESQFEVRKVVE--- 585

PsToc159 AESNVDRVGEVEDDT--------------HFD-NAVEEEAE--------SNVDRVVEVED 569

Solyc09g074940.1.1 VVAVTEEIKEADPET-----------VNKSLDTKDVEVEPE--------QAVSGTIYAN- 494

. . . : : .

OsToc159 REEDAD-DDEEIVLAAA----------DDEDDGTNEADD------DEDGVSSDRGPARVA 448

SbToc159 REEEAA-DNDII-EVVP----------DDEDGVGNEADD------DDDGANSDTSPARVA 484

AtToc159 GDSAEEDENKLPVEDIV----SSREFSFGGKEVDQ--EPSGEGVTRVDGSES-EEETEEM 638

PsToc159 DTHFDN-----AVEEEADSNVDRVIEMDDGSHVEAAVDHHID--REIDDLLS-DSKDESM 621

Solyc09g074940.1.1 GDHSGESVERDVVEVEVSGQTSAISRSITGSEQEGEAKDHIDEEANLEGSVS-DGETDGM 553

. . :. *

OsToc159 IIESSEAAKQIMKELGEGSASVSPVSG-LSSSREYTNSMDGQIVLDDSEEDGDDDDNEDD 507

SbToc159 ILESSEAAKQIMKELAEGSSG--------SVSRDFTNSMDGQIMLDDSEDDEDDDDNDDS 536

AtToc159 IFGSSEAAKQFLAELEKASSGIEAHSDEANISNNMSDRIDGQIVTDSDEDVD--TEDEG- 695

PsToc159 IFGGSDSANKYLEELEKQIRDSE---------SSQGDRIDGQIVTDSDEEDV--SDEEGG 670

Solyc09g074940.1.1 IFGSSEAAKQFMEELERESGGGSY------AGAEVSQDIDGQIVTDSDEEAD--TDEEGD 605

*: .*::*:: : ** . . . : :****: *..*: :::.

OsToc159 DEKGFDSAALAALLKAATGA--SADGNVTVSSQDGSRIFSMDRPAGLGSSAPSLRPTVPR 565

SbToc159 DEKGFDSAALAALLKAATGG--SSDGNITVASPDGSRIFTMDRPAGLGSSAPSLRPTAPR 594

AtToc159 EEKMFDTAALAALLKAATGGGSSEGGNFTITSQDGTKLFSMDRPAGLSSSLRPLKPAAAP 755

PsToc159 SKELFDTATLAALLKAASGAGGEDGGGITLTAQDGSRLFSVERPAGLGPSLQTGKPAV-- 728

Solyc09g074940.1.1 VKELFDSAALAALLKAATG-GDSDGGNITVTSQDGSRLFSVERPAGLGSSLRSLRPAP-- 662

:: **:*:********:* . .*..*::: **:::*:::*****. * :*:

OsToc159 PVARSNLFSPSELAV-TAEPTEEMTEEEKKLHDKVELIRVKFLRLVYRLGATPEETVAAQ 624

SbToc159 QPARSNLFSPSELAV-TADPTEEMTEEEKKLHDKVELIRVKFLRLVYRLGATPEETVAAQ 653

AtToc159 RANRSNIFSNSNVTM-ADETEINLSEEEKQKLEKLQSLRVKFLRLLQRLGHSAEDSIAAQ 814

PsToc159 RSIRPNLFAPSMSRAGTVVSDTDLSEEDKKKLEKLQEIRIKYLRVIQRLGFTTEESIAAQ 788

Solyc09g074940.1.1 RPSQPNLFTHSNLQN-SGESENNLSEEEKKKLDTLQQIRVKFLRLIHRLGLSSDEPIAAQ 721

: *:*: * : :::**:*: :.:: :*:*:**:: *** : :: :***

OsToc159 VLYRLSLAEGIRHGRQTNRAFSLDNARKKAMLLEAEGKEELNFSCNILVLGKIGVGKSAT 684

SbToc159 VLYRLSLAEGIRHGRQTNRAFSLDNARRKALLLEAEGKEELNFSCNILVLGKTGVGKSAT 713

AtToc159 VLYRLALLA----GRQAGQLFSLDAAKKKAVESEAEGNEELIFSLNILVLGKAGVGKSAT 870

PsToc159 VLYRLTLVA----GRQIGEMFSLDAAKESASRLEAEGRDDFAFSLNILVLGKTGVGKSAT 844

Solyc09g074940.1.1 VLYRMTLIA----RRQNSPLFSVEAAKMKAFQLEAEGKDDLDFSVNILVIGKSGVGKSAT 777

****::* ** . **:: *: .* ****.::: ** ****:** *******

OsToc159 INSIFGEEKSKTDAFSSATNSVREIVGNVDGVQIRIIDTPGLRPNVMDQGSNRKILASVK 744

SbToc159 INSIFGEEKSKTDAFSSATTNVREIIGDVDGVKIRIIDTPGLRPNVMDQGSNRKILAAVK 773

AtToc159 INSILGNQIASIDAFGLSTTSVREISGTVNGVKITFIDTPGLKSAAMDQSTNAKMLSSVK 930

PsToc159 INSIFGETKTSFSAYGPATTSVTEIVGMVDGVEIRVFDTPGLKSSAFEQSYNRKVLSTVK 904

Solyc09g074940.1.1 INSIFGEEKTSIDAFGPATTSVKEISGVVDGVKIRVFDTPGLKSSAMEQGFNRSVLSSVK 837

****:*: :. .*:. :*..* ** * *:**:* .:*****: .::*. * .:*::**

OsToc159 KYTKRCPPDIVLYVDRLDSLSRDLNDLPLLKTITSVLGSSIWFNAIVALTHAASAPPEGL 804

SbToc159 KYTKKCPPDIVLYVDRLDSLSRDLNDLPLLKTITAVLGSSIWFNAIVALTHAASAPPEGL 833

AtToc159 KVMKKCPPDIVLYVDRLDTQTRDLNNLPLLRTITASLGTSIWKNAIVTLTHAASAPPDGP 990

PsToc159 KLTKKSPPDIVLYVDRLDLQTRDMNDLPMLRSVTSALGPTIWRNVIVTLTHAASAPPDGP 964

Solyc09g074940.1.1 KLTKKNPPDIYLYVDRLDAQTRDLNDLPMLKTITSCLGPSIWRSAIVTLTHGASAPPDGP 897

* *: **** ******* :**:*:**:*:::*: ** :** ..**:***.*****:*

OsToc159 NGAPMTYEVLMAQRSHIIQQSIRQAAGDMRLM-----NPVALVENHPSCRRNREGQKVLP 859

SbToc159 NGAPMTYEVLMAQRSHIIQQSIRQAAGDMRLM-----NPVALVENHPSCRKNREGQKVLP 888

AtToc159 SGTPLSYDVFVAQCSHIVQQSIGQAVGDLRLMNPSLMNPVSLVENHPLCRKNREGVKVLP 1050

PsToc159 SGSPLSYDVFVAQRSHIVQQAIGQAVGDLRLMNPNLMNPVSLVENHPSCRKNRDGQKVLP 1024

Solyc09g074940.1.1 SGSPLSYEVFVTQRSHVVQQSIGQAVGDLRMMSPSLMNPVSLVENHPSCRRNRDGHKILP 957

.*:*::*:*:::* **::**:* **.**:*:* ***:****** **:**:* *:**

OsToc159 NGQSWRHQMLLLCYSSKILSEANSLLKLQDPN-PGKLFGFRFRSPPLPFLLSSLLQSRAH 918

SbToc159 NGQSWRHQMLLLCYSSKILSEANSLLKLQDPN-PGKLFGFRFRSPPLPFLLSSLLQSRAH 947

AtToc159 NGQTWRSQLLLLCYSLKVLSETNSLLRPQEPLDHRKVFGFRVRSPPLPYLLSWLLQSRAH 1110

PsToc159 NGQSWKPLLLLLCYSMKILSEATNISKTQEAADNRRLFGFRSRAPPLPYLLSWLLQSRAH 1084

Solyc09g074940.1.1 NGQSWRPQLLLLSYSMKILSEASALSKPEDPFDHRKLFGFRTRSPPLPYMLSSMLQSRAH 1017

***:*: :***.** *:***:. : : :: ::**** *:****::** :******

OsToc159 PKLSPDQGGNEGDSDIDLDDYSDIEQDEDE-EEYDQLPPFKPLTKSQLARLTKEQKNAYF 977

SbToc159 PKLSAEQGGNEGDSDIELDDYSDVEQDDDE-EEYDQLPPFKPLTKAQLARLTKEQKNAYF 1006

AtToc159 PKLPGDQGGDSVDSDIEIDDVSDSEQEDGEDDEYDQLPPFKPLRKTQLAKLSNEQRKAYF 1170

PsToc159 PKLPDQAGIDNGDSDIEMADLSDSDGEEGE-DEYDQLPPFKPLKKSQIAKLNGEQRKAYL 1143

Solyc09g074940.1.1 PKLSAEQGGDNGDSDIDLDDLSDSDQE-EE-DEYDQLPPFKPLRKAQLAKLSKEQRKAYF 1075

*** : * :. ****:: * ** : : * :*********** *:*:*:*. **::**:

OsToc159 DEYDYRVKLLQKKQWKDEIRRLKEMKKRGKTDM-DAYGYANIAGENDLDPPPENVSVPLP 1036

SbToc159 DEYDYRVKLLQKKQWKDEIRRLKEMKKRGKTDL-DDYGYASIGGENDQDPPPENVSVPLP 1065

AtToc159 EEYDYRVKLLQKKQWREELKRMKEMKKNGKKLGESEFGYPGEED-DPENGAPAAVPVPLP 1229

PsToc159 EEYDYRVKLLQKKQWREELKRMRDMKKRGKNG---ENDYM--EE-DEENGSPAAVPVPLP 1197

Solyc09g074940.1.1 EEYDYRVKLLQKKQLREDLKRMKEMKSKGKEAA-IDNGYA--EE-EADAGAAAPVAVPLP 1131

:************* :::::*:::**..** .* : * ****

OsToc159 DMVLPPSFDCDNPTYRYRFLEPTSTVLARPVLDAHGWDHDCGYDGVSVEETLALLNKFPA 1096

SbToc159 DMVLPPSFDCDNPTYRYRFLEPTSTVLARPVLDAHGWDHDCGYDGVSVEETLAILSRFPA 1125

AtToc159 DMVLPPSFDSDNSAYRYRYLEPTSQLLTRPVLDTHGWDHDCGYDGVNAEHSLALASRFPA 1289

PsToc159 DMVLPQSFDSDNPAYRYRFLEPNSQLLTRPVLDTHSWDHDCGYDGVNIENSMAIINKFPA 1257

Solyc09g074940.1.1 DMALPPSFDSDNPAYRYRFLEPTSQFLARPVLDTHGWDHDCGYDGVNVEQSLAIASRFPA 1191

**.** ***.** :****:***.* .*:*****:*.**********. *.::*: .:***

OsToc159 NMAVQVTKDKKEFSIHLDSSISAKLGEDASSLAGFDIQTVGRQLAYILRGETKFKNIKKN 1156

SbToc159 NVAVQVTKDKKEFSIHLDSSIAAKHGENASSLAGFDIQTVGRQLAYILRGETKIKNIKKN 1185

AtToc159 TATVQVTKDKKEFNIHLDSSVSAKHGENGSTMAGFDIQNVGKQLAYVVRGETKFKNLRKN 1349

PsToc159 AVTVQVTKDKQDFSIHLDSSVAAKHGENGSTMAGFDIQNIGKQLAYIVRGETKFKNFKRN 1317

Solyc09g074940.1.1 AVTVQITKDKKDFSINLDSSIAAKHGENGSTMAGFDIQSIGKQLAYIVRGETKFKSLKKN 1251

:**:****::*.*:****::** **:.*::******.:*:****::*****:*.:::*

OsToc159 KTTGGFSVTFLGDIVATGLKVEDQLSLGKRLALVASTGAMRAQGDTAYGANLEARLKDKD 1216

SbToc159 KTTGGFSVTFLGDIVATGLKVEDQLSLGKRLSLVASTGAMRAQGDTAYGANLEARLKDKD 1245

AtToc159 KTTVGGSVTFLGENIATGVKLEDQIALGKRLVLVGSTGTMRSQGDSAYGANLEVRLREAD 1409

PsToc159 KTAAGVSVTFLGENVSTGVKLEDQIALGKRLVLVGSTGTVRSQNDSAYGANVEVRLREAD 1377

Solyc09g074940.1.1 KTACGISVTFLGENMVTGLKVEDQIILGKQYVLVGSAGTVRSQSDTAYGANFELQRREAD 1311

**: * ******: : **:*:***: ***: **.*:*::*:*.*:*****.* : :: *

OsToc159 YPIGQSLSTLGLSLMKWRRDLALGANLQSQFSIGRGSKMVVRLGLNNKLSGQITVRTSTS 1276

SbToc159 YPIGQSLSTLGLSLMKWRRDLALGANLQSQFSIGRGSKMAVRLGLNNKLSGQITVRTSTS 1305

AtToc159 FPIGQDQSSFGLSLVKWRGDLALGANLQSQVSVGRNSKIALRAGLNNKMSGQITVRTSSS 1469

PsToc159 FPVGQDQSSLSLSLVQWRGDLALGANFQSQISLGRSYKMAVRAGLNNKLSGQINVRTSSS 1437

Solyc09g074940.1.1 FPIGQVQSTLSMSVIKWRGDLALGFNSMAQFAVGRNSKVAVRAGINNKLSGQVTVRTSSS 1371

:*:** *::.:*:::** ***** * :*.::**. *:.:* *:***:***:.****:*

OsToc159 EQVQIALLGLIPVAASIYRSFRPSEPSFAY--- 1306

SbToc159 EQVQIALLGLIPVAASIYRSFRPSEPSFAY--- 1335

AtToc159 DQLQIALTAILPIAMSIYKSIRPEATNDKYSMY 1502

PsToc159 DQLQIALIAILPVAKAIYKNFWPGVT-ENYSIY 1469

Solyc09g074940.1.1 DHLSLALTAIIPTAIGIYRKLWPDAG-EKYSIY 1403

:::.:** .::* * .**:.: * *

Figure 1E –Source data:

Alignment of the amino acid sequences of *Arabidopsis* (At) TOC159 with following species: *Pisum sativum* (Ps)*, Solanum lycopersicum* (Sl), *Oryza sativa* (Os), and *Sorghum bicolor* (Sb). Sequence alignment was carried out using CLUSTAL Omega (1.2.4). The conserved SUMOylation sequence in the M-domain marked in the red box.
