# Supplementary material for: SUMOylation contributes to proteostasis of the chloroplast protein import receptor TOC159 during early development
Source: eLife. 2020 Dec 22;9:e60968. doi: 10.7554/eLife.60968 (PMC8497054; doi:10.7554/eLife.60968)
Supplement: Supplementary file 1. [file elife-60968-supp1.docx]

**Supplementary file 1.** List of primers used in this study.

| **Primer name** | **Sequence (5'-3')** |
| --- | --- |
| S1-F | CCTTTCCATATGTCTGCAAACCAGGAGG |
| S1-R | CCTGGACGAATTCTCAGGCCGTAGCACC |
| S2-F | GATAATAAAGTCATATGTCTGCTACTCCG |
| S2-R | CCATTAAAATAAGAATTCCTAAAAGCAGAAGAGC |
| S3-F | GATAAAAGGACATATGTCTAACCCTCAAGATG |
| S3-R | CCAAATTATGAATTCTTAAAGCCCATTATGATGG |
| SUMO3–F(GATE) | GGGGACAAGTTTGTACAAAAAAGCAGGCTTCATGTCTAACCCTCAAGATGACAAGCC |
| SUMO3–R(GATE) | GGGGACCACTTTGTACAAGAAAGCTGGGTCCTAAAGCCCATTATGATGGAAAAGC |
| TOC159S3F | GCCACTGGGGTCCGACTCGAGGACCAAATAGC |
| TOC159S3R | GCTATTTGGTCCTCGAGTCGGACCCCAGTGGC |
| TOC159 INT | GAATAGGGTTTTAATCGGAAG |
| TOC159 A3R | TGCCACATCAACATGCACTG |
| LB5 | GATGCAATCGATATCAGCCAATTTTAGAC |
